# A Nanosensor Based on a Metal-Insulator-Metal Bus Waveguide with a Stub Coupled with a Racetrack Ring Resonator

**DOI:** 10.3390/mi12050495

**Published:** 2021-04-27

**Authors:** Haoran Shi, Shubin Yan, Xiaoyu Yang, Xiushan Wu, Wenchang Wu, Ertian Hua

**Affiliations:** 1 School of Instrument and Electronics, North University of China, Taiyuan 030051, China; S1906046@st.nuc.edu.cn (H.S.); S1806069@st.nuc.edu.cn (X.Y.); 2 School of Electronical Engineering, Zhejiang University of Water Resources and Electric Power, Hangzhou 310058, China; wuxs@zjweu.edu.cn (X.W.); huaet@zjweu.edu.cn (E.H.); 3 Zhejiang-Belarus Joint Laboratory of Intelligent Equipment and System for Water Con-Servancy and Hydropower Safety Monitoring, Hangzhou 310018, China; shihr@zjweu.edu.cn; 4 School of Electron and Information Engineering, Ningbo University of Technology, Ningbo 310058, China

**Keywords:** MIM, racetrack ring resonator, Fano resonance, EIT-like, nanosensor

## Abstract

A nanostructure comprising the metal-insulator-metal (MIM) bus waveguide with a stub coupled with a racetrack ring resonator is designed. The spectral characteristics of the proposed structure are analyzed via the finite element method (FEM). The results show that there is a sharp Fano resonance profile and electromagnetically induced transparency (EIT)-like effect, which are excited by a coupling between the MIM bus waveguide with a stub and the racetrack ring resonator. The normalized H_Z_ field is affected by the displacement of the ring from the stub *x* greatly. The influence of the geometric parameters of the sensor design on the sensing performance is discussed. The sensitivity of the proposed structure can reach 1774 nm/RIU with a figure of merit of 61. The proposed structure has potential in nanophotonic sensing applications.

## 1. Introduction

Surface plasmon polaritons (SPPs) are a kind of electromagnetic phenomenon and are bound to the vertical direction of propagation in the form of exponential decay. SPPs are derived from the free electrons on the surfaces of metals (or doped semiconductor materials, polar materials, two-dimensional materials, etc.) that exhibit metallicity [1]. Under the excitation of incident light, the movement of electrons and the incident photoelectric magnetic field excite each other, and a collective resonant motion occurs. This can be compared with quantized quasiparticles and phonons in the collective resonance motion of atoms in a crystal. SPPs are the quasiparticle description of the collective resonance motion of electrons on a metal surface [2]. The excitation of surface plasmons will inevitably lead to strong resonance absorption or scattering of incident photons of specific frequencies (energy) on the metal surface, which can focus the energy of the electromagnetic field to a range of subwavelength scale, causing a strong light field enhancement effect, which greatly increases the interaction between light and matter [3]. SPPs have achieved outstanding development in multidisciplinary basic research and cross-applications [4].

Fano resonance was discovered and proposed by the Italian physicist U. Fano in 1961 [5]. In the atomic system, it appears as a quantum interference phenomenon caused by the interaction of a Lorentz narrow discrete state and a broad continuous state, and zero absorption occurs at a specific optical frequency, resulting in asymmetrical spectral lines [6,7]. As the curve of Fano resonance is asymmetric and steep, it has a high space electromagnetic field constraint ability, of which the subtle wavelength shift is easy to distinguish [8]. The MIM waveguide structure, geometric size, and small changes in the surrounding environment will make the transmittance and the position of Fano resonance peak or dip change significantly [9].

Electromagnetically induced transparency (EIT) is a kind of quantum interference effect in the atomic field. The interference effect between the ground state to the excited state and the excited state to the metastable state results in a narrow transparent window in the wide absorption spectrum [10]. EIT in photonics is a coherent optical nonlinearity that makes the medium transparent in a narrow spectral range around the absorption lines. In the transparent window, the dispersion characteristics change greatly, which causes the EIT effect that has a good application prospect in slow light devices, nonlinear optics, optical storage, optical switches, etc. [11]. As a kind of similar coherent effect, the EIT-like effect has attracted extensive attention in recent years and has been proved in classical systems such as surface plasmonic devices, photomechanical systems, coupled optical resonators, and metamaterial configurations [10]. Especially in various types of resonant systems, such as microring resonators, microsphere resonators, photonic crystal resonators and isoplason resonators, by designing coherent excitation paths, the interference phenomena similar to the EIT effect can be realized [10,12].

Compared with discrete and continuous states without coupling effects, the Fano resonance effect has stronger near-field local characteristics, a higher resolution, narrower full width at half maximum (FWHM) [13,14,15] and is suitable for light transmission and control at subwavelength sizes [16,17]. Its resonance characteristics have been widely used in surface enhanced Raman scattering, wavelength division multiplexing, refractive index sensing and optical switching. Therefore, many nanosensors based on the Fano resonance have been proposed [18,19]. Chen et al. designed a surface plasmon waveguide structure and the sensitivity is up to 1180 nm/RIU [20]. Qi et al. presented an asymmetric plasmonic resonator system with a sensitivity of up to 1350 nm/RIU [21]. Yang et al. designed a MIM waveguide structure with a sensitivity of 1075 nm/RIU [22]. Wang et al. designed an isosceles triangular cavity with a sensitivity of about 1200 nm/RIU [23]. Additionally, Zhu et al. proposed a key-shaped cavity achieving a sensitivity of 1261.67 nm/RIU [24]. Butt et al. proposed a Nanodots decorated asymmetric metal-insulator-metal waveguide resonator structure with a sensitivity of 2464 nm/RIU [25]. Chao et al. proposed a MIM waveguide with a side-coupled ring with several silver rods with a high sensitivity of 2080 nm/RIU [26].

In addition to the design we mentioned above, other approaches for designing the refractive index sensor and SPPs in the MIM waveguides have been studied. Recently, some plasmonic RI sensors using all-metal nanostructures have been proposed. Due to the nanorod arrays being easy to link with the sensing medium compared to the metal-dielectric-metal structures for detecting and sensing applications, numerous plasmonic metal nanorod structures have been explored for the purpose of absorption and they are based on SPR and GPR effects. Although there have been a number of articles on diverse plasmonic MIM waveguides, the interactions of incident Mid-infrared (MIR) wave and tunable MIM waveguides are investigated less. The MIR spectrum is in the wavelength range of 2 to 20 µm, which represents the molecular fingerprint zone, and the potential MIR applications have been widely reported in many works. In particular, plasmonic MIM waveguide sensors are necessary for an atmospheric transparent window of the MIR spectrum from 2 to 12 µm. Therefore, there are still many efforts in this field that need to be made [27,28,29].

In this study, a MIM waveguide structure comprising the MIM bus waveguide with a stub coupled with a racetrack ring resonator was proposed and analyzed. The effects of different geometric parameters on the normalized field distributions H_Z_, transmission responses, the sensitivity (S) and figure of merit (FOM) were studied in depth.

## 2. Materials and Methods

**Figure 1:**
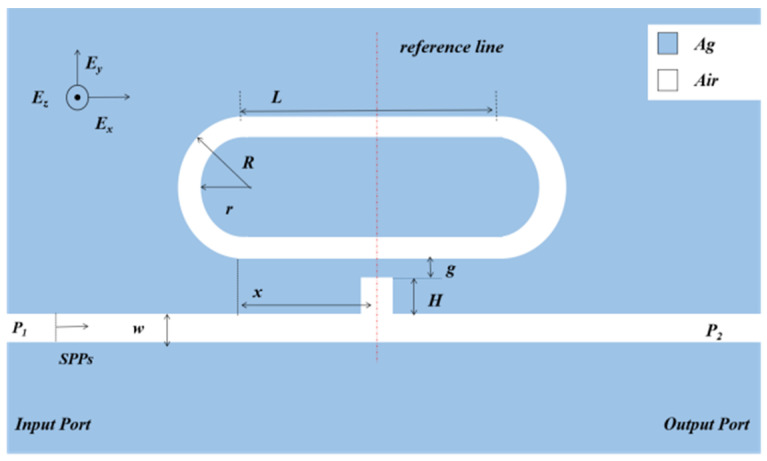
The geometric structure of the coupled racetrack ring resonator.

Figure 1 represents the graphical illustration of the proposed sensor design comprising a racetrack ring resonator coupled with a MIM bus waveguide with a stub. Here, we used the 2D model to replace the 3D one, which can simplify the calculation. If the structure height is larger than the skin depth of SPPs (in real photonic devices, the structure height is larger than the skin depth of SPPs), the role of structure height in the losses of the 3D model can be approximated by a 2D model [30,31]. The material losses have a minute variation as the height of the waveguide is greater than 200 nm, which shows that the height of the waveguide does not affect material losses after a certain value. Even if the height of the waveguide approaches infinity, the material losses stay the same, which is the case of a 2D simulation [32]. The blue and white parts represent the Ag layer and air ($\varepsilon_{S}$ = 1), respectively. The transmission spectra were obtained by the numerical FEM, and the dieletric of Ag can be expressed using the Debye–Drude dispersion model:

(1) $$\varepsilon_{\mathrm{Ag}}(\omega )=\varepsilon_{1}(\omega )+\varepsilon_{2}(\omega )i=1-\frac{\omega_{p}^{2}\tau^{2}}{1+w^{2}\tau^{2}}+(1-\frac{\omega_{p}^{2}\tau }{\omega (1+w^{2}\tau^{2})})i$$

where *ω*, *ω_p_* = 1.38 × 10^16^ rad/s and τ = 7.35 × 10^−15^ s represent the angular frequency of the light, the plasma frequency of Ag and the relaxation time, respectively [6,8,14]. The Drude model is the most classical model for calculating the dielectric constant. It fits well with the experimental data at short wavelengths, but it causes large errors at long wavelengths. Therefore, it is necessary to introduce the Lorentz model to modify it. The Lorentz model takes into account the influence of the transition between bands, but the calculation is more complicated. Therefore, we adopted the Debye–Drude model: the dielectric constant of the medium is a parameter related to frequency. To express this frequency dependence of the medium, the most commonly used model is the Debye model [33,34,35].

We chose the racetrack ring resonator as our structure mainly based on the following aspects: it combines the advantages of the circle [26], rectangle [36] and ellipse [37] resonators. The circle resonator is the best choice of resonator. However, if the radius is too large, its FWHM will increase, and the sensing performance will decrease. The straight waveguide in the rectangle is very conducive to light transmission, but it will cause a larger size at four right angles. The transmission loss of the ellipse can increase the sensing area, but its radius of curvature will also cause much material loss, so we chose the racetrack ring resonator.

The length of the racetrack ring resonator is denoted as *L*. The outer and inner radii of the racetrack ring resonator are described as *R* and *r*. The height of the stub is denoted by *H*. *x* is the displacement of the ring from the stub. *g* is defined as the coupling distance. In this study, we set *w*, which stands for the width of the MIM bus waveguide, at 50 nm to ensure only TM_0_ modes exist in the waveguide. *P_1_* and *P_2_* are the input port and output port, respectively. SPPs are excited by incident photons coupled with free electrons on the surface of Ag.

Simulation is a good method to study and optimize SPP structures due to their size. The physical model was built by the COMSOL Multiphysics 5.4 software, which can also obtain the transmission spectrum by the FEM. The absorption boundary condition was set as the perfect matched layer and ultrafine meshing was chosen to ensure calculation accuracy.

## 3. Results

**Figure 2:**
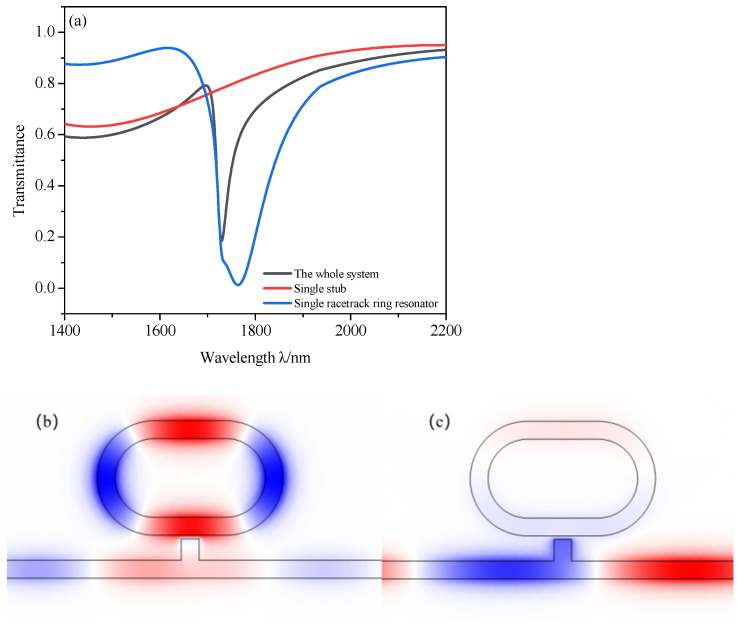
(**a**) Transmission spectra of the whole system, single stub and single racetrack ring resonator; (**b**,**c**) the magnetic field distribution H_Z_ at λ = 1729 and 2000 nm, respectively.

To understand the transmission properties of the designed structure, firstly, the whole system, single stub and single racetrack ring resonator were compared. We fixed the initial structural parameters as *L* = 200 nm, *h* = 100 nm, *R* = 140 nm, *r* = 90 nm, *g* = 10 nm, *x* = 100 nm. The results are plotted in Figure 2 and the transmittance T = *P_2_*/*P_1_* [38].

Figure 2a shows that the whole system exhibits an obvious Fano resonance curve. The single stub excites a broad spectrum resonance mode. The single racetrack ring resonator excites a narrow spectrum resonance mode (here, we set *g* as 10 nm). Therefore, the Fano resonance is excited by the coupling between different resonance modes of the whole system. For the sharp and asymmetric line shape, the transmittance can increase from the dip to the peak more rapidly than in the conventional resonators with symmetric Lorentz-like line resonant spectra. This can significantly reduce the required wavelength shift for the plasmonic modulators as well as improve the wavelength resolution for the plasmonic splitters and demultiplexers [39].

The dip of the transmission wavelength is denoted by the incident wavelength, which ($\lambda_{FR}$) can be calculated, according to the standing wave theory, by:

(2) $$\lambda_{FR}=\frac{2L_{eff}n_{eff}}{f-\varphi_{ref}/\pi }\;$$

(3) $$n_{eff}={\left[\varepsilon_{\mathrm{Ag}}+{\left(k/k_{0}\right)}^{2}\right]}^{1/2}$$

where *L_eff_* is the propagation length of SPPs in the cavity, $\varphi_{ref}$ is the phase shift of SPP reflection in the cavity, *n_eff_* is the effective refractive index. Additionally, *f* is the resonance mode order (*f* = 1, 2, 3, …) [15]. *k* and *k_0_* are the wave vector in the waveguide and wave vector in free space and *k* = $\sqrt{\beta^{2}-\varepsilon_{\mathrm{Ag}}k_{0}^{2}}$, *k_0_* = 2π/λ*_FR_*, *β* is the propagation constant [5,10,20].

In order to further understand the underlying principle of Fano resonance, the H_Z_ field distributions are studied and plotted in Figure 2b,c. From Figure 2b, the power flow is almost concentrated in the racetrack ring resonator, and the reason for which is that destructive interference is induced between the incident SPPs and the SPPs from the stub to MIM bus waveguide. From Figure 2c, the power flow is almost concentrated in the MIM bus waveguide and transmission is enhanced, the reason for which is that constructive interference is induced between the two excitations [36].

**Figure 3:**
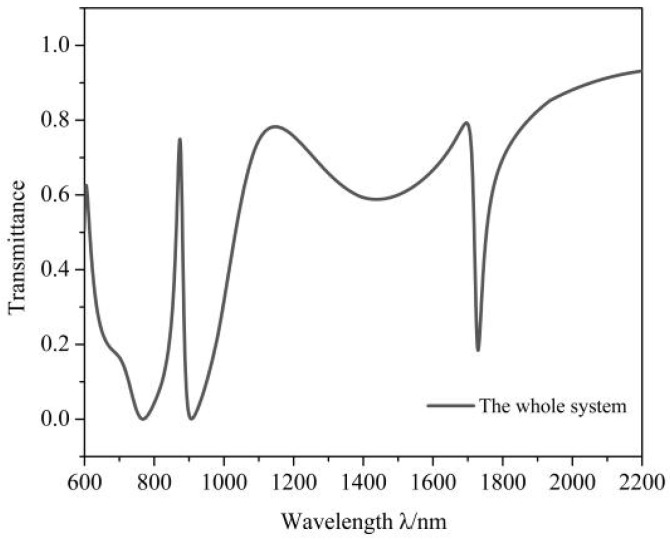
Transmission spectrum of the system.

As shown in Figure 3, there is a Fano resonance dip and an EIT-like peak in the transmission spectra and they are described as *F_1_* (λ = 1729 nm) and *F_2_* (λ = 835 nm). EIT-like, a special Fano resonance, is excited by a coupling between a narrow band, corresponding to the MIM bus waveguide with a stub, and a broad band corresponding to the racetrack ring resonator when the dip of narrow band exists in the dip of broad band.

The FWHM of *F_2_* is narrower than that of *F_1_*, but the sensitivity of *F_2_* getting 840 nm/RIU lower than that of *F_1_* that is 1320 nm/RIU. Therefore, we choose *F_1_* as the object of this study.

According to Equations (1)–(3) and the effective SPPs wavelength, λSPPs=λFR/Re(neff), when $\lambda_{\mathrm{FR}}$ = 1729 nm, $\lambda_{\mathrm{SPPs}}$ = 1235 nm, $(2\pi R+2L)/\lambda_{\mathrm{SPPs}}\approx 1$, which shows that $\lambda_{\mathrm{SPPs}}$ = 1235 nm meets the condition of Fano resonance and conforms to the simulation result when *f* = 1 [10].

The S and FOM are important values for a sensor, which are described as:

(4) $$S=\frac{\Delta \lambda }{\Delta n}$$

(5) $$FOM=\frac{S}{FWHM}$$

where Δλ is the shift of the resonance wavelength; Δn is the change in refractive index [10,12,17].

**Figure 4:**
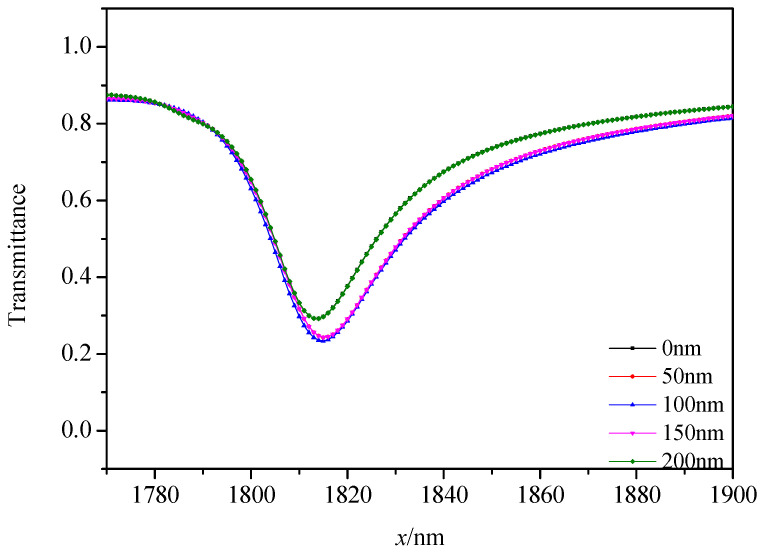
Transmission spectra of different displacements of the ring from the stub *x*.

We then analyzed the effects of different similar structures on the transmission spectra and normalized magnetic field H_Z_. The magnetic field enhancements mainly arose from the superposition of the incident SPPs with the reflected SPPs at the boundary of the air and metal intersurface structure. This phenomenon is important for the design of plasmonic sensors where larger magnetic field intensities are desired [40]. The structure parameters are fixed as *L* = 200 nm, *h* = 100 nm, *R* = 140 nm, *r* = 90 nm, *g* = 10 nm, except the displacement of the ring from the stub *x*. We studied the transmission spectra of different structures, whose x values are 0, 50, 100, 150 and 200 nm, respectively. As depicted in Figure 4, there are differences among the transmittance spectra of these five structures. Some observable phenomena can be seen in Figure 4, when *x* = 100 nm, which means that when the stub is in the central of the system, the transmittance is as its lowest. When the stub moves to *x* = 50 and 150 nm, the transmittance increases. When the stub moves to *x* = 0 and 200 nm, the transmittance is the highest. We found that when the distance of the stub to reference line is the same, the transmittance is similar. As for *x* = 0 and 200 nm, their transmission spectra have the narrowest FWHM among these structures but a high transmittance, which leads to poor sensing properties and is difficult to detect at *P_out_*. When *x* = 100 nm, the structure had the most obvious Fano resonance and lowest transmittance, which led to the best sensing properties of these structures. At the same time, the $\lambda_{FR}$ was almost unchanged when *x* was changed.

**Figure 5:**
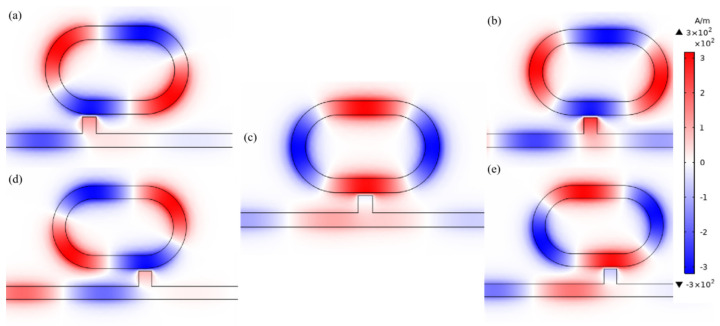
The magnetic field distribution H_Z_ at the Fano resonance dip λ = 1815 nm of (**a**) *x* = 0 nm; (**b**) *x* = 50nm; (**c**) *x* = 100 nm; (**d**) *x* = 150 nm; (**e**) *x* = 200 nm.

Furthermore, the normalized H_Z_ field of these structures was analyzed, as plotted in Figure 5. We can clearly observe in Figure 5 that when the displacement (*x*) of the ring from the stub was changed, the symmetry axes of the normalized H_Z_ field of these structures rotated around the center point of the system. Especially, when *x* = 100 nm, the near-field intensity pattern strongly localized at the racetrack ring resonator with respect to H_Z_ at other structures, which can be seen from the effectively localized magnetic field. The discrepancy of the resonance dip at a different transmittance is due to the different coupling position where different interferences of near-field coupling effect [40] and phase coupling effect [40] occur between the incident SPPs and the racetrack ring resonator. According to the above analysis, we can see that we can change *x* to obtain different transmittances and a normalized H_Z_ field. Hence, we chose an *x* = 100 nm structure to study in this paper.

**Figure 6:**
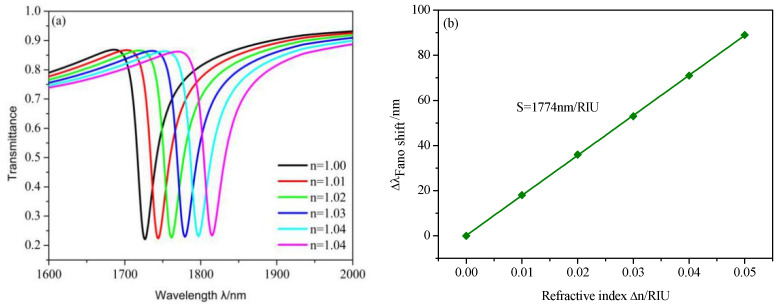
(**a**) Transmission spectra of the refractive indices ranging from 1.00 to 1.05 RIU. (**b**) Linear fitting of the sensitivity.

The measuring principle of the plasmonic refractive index sensing structure is that the resonance dip will shift with the changing of the refractive index of surrounding materials. The refractive index *n* varies from 1.00 to 1.05 RIU in a step of 0.01 RIU, with the default structure parameters *L*, *R*, *r*, *h*, *x* and *g* being set at 200, 160 110, 60, 100 and 10nm. It is obvious in Figure 6a that the transmission spectra show a remarkable blueshift with the increasing *n*. Figure 6a shows that the Fano resonance dip has a nearly linear shift with the change in Δn. From Figure 6b, the *S* is 1774 nm/RIU and the FOM is 61 when the FWHM is 29. The default values are the best structure parameters and the properties are better than most of those listed in Table 1.

As the size of nanostructure is small and the working wavelength is about 1815 nm, the material loss must be taken into account. When $\omega <\omega_{p}$, the imaginary part of $\varepsilon_{Ag}(\omega )$ should be taken into consideration, which stands for the absorption loss. The *tg* (ε(ω)) is the general formula of dielectric loss, and was calculated at about 0.12, which is a reasonable value. The skin depth of silver is described as $\delta_{Ag}=\lambda /(4\pi \sqrt{\left|\varepsilon (\omega )\right|})$, which is 0.77 nm in this system. Additionally, this means that the SPPs are bound to the metal surface.

**Figure 7:**
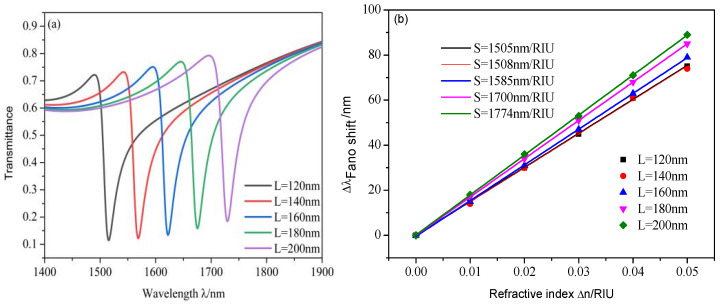
(**a**) Transmission spectra of the different length of the racetrack ring resonator *L*; (**b**) linear fitting of the sensitivity.

Subsequently, the effects of geometric parameters of the design on sensing performance were studied. The length of the racetrack ring resonator *L* is discussed first. The value of *L* changes from 120 to 200 nm at intervals of 20 nm and other values are set as default values when *n* = 1, whose results are shown in Figure 7. The resonance dip shows an obvious redshift with an increasing *L,* as depicted in Figure 7a, which can be explained by Equation (2): when *L* increases, the propagation length of SPPs in the Fano resonance cavity will increase, which will cause the value of $\lambda_{FR}$ to increase. Moreover, it is clear that with increasing *L* the transmittance increases, but the *S* of the design will increase from 1505 to 1774 nm/RIU when *L* increases from 120 to 200 nm, as plotted in Figure 7b.

**Figure 8:**
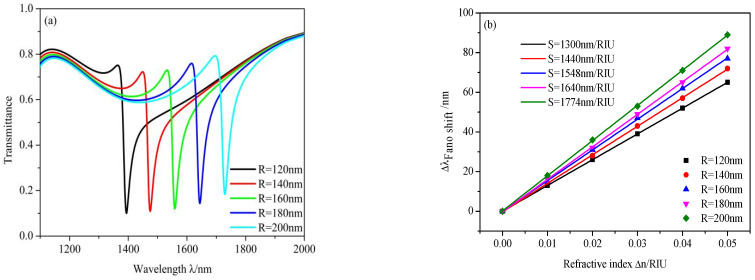
(**a**) Transmission spectra of the different radius of the racetrack ring resonator *R*; (**b**) linear fitting of the sensitivity.

Then, the effects of the outer radius of racetrack ring resonator *R* were analyzed. The values of *R* changed from 120 to 160 nm at intervals of 10 nm and other values were set as default values when *n* = 1, whose results are shown in Figure 8. The resonance dip shows an obvious redshift with increasing *R* as depicted in Figure 8a, which can also be explained by Equation (2) as *L* could. The *S* of the design increases from 1505 to 1774 nm/RIU when *R* increases from 120 to 200 nm, as plotted in Figure 8b. Therefore, the length of the racetrack ring resonator *L* and the outer radius of the racetrack ring resonator *R* influences not only the position of Fano dip but also *S*, which correspond to the narrow band of this system.

**Figure 9:**
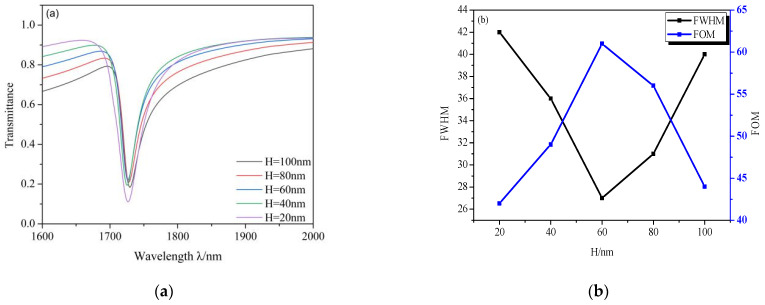
(**a**) Transmission spectra of *H* varying from 20 to 100 nm; (**b**) the tendency of FWHM and FOM with increasing *H*.

Furthermore, we analyzed the influence of different *H* values on the transmission properties. We changed the values of *H* from 20 to 100 nm in a step of 20 nm and other values were set as default values when *n* = 1, whose results are shown in Figure 9. It is clear that by increasing *H* from 20 to 60 nm, the FWHM decreases, which means that FOM increases and, when increasing *H* from 60 to 100 nm, the FWHM increases, which means that the FOM decreases. The FOM of the design increases from 42 to 61 and from 61 to 44, as depicted in Figure 9b. However, when *H* increases, it can be seen that the transmittance increases obviously, which means that *H* corresponds to the broad band in this system, and results in weakening the sensing performance. Considering these, we set *H* as 60 nm in this study.

**Figure 10:**
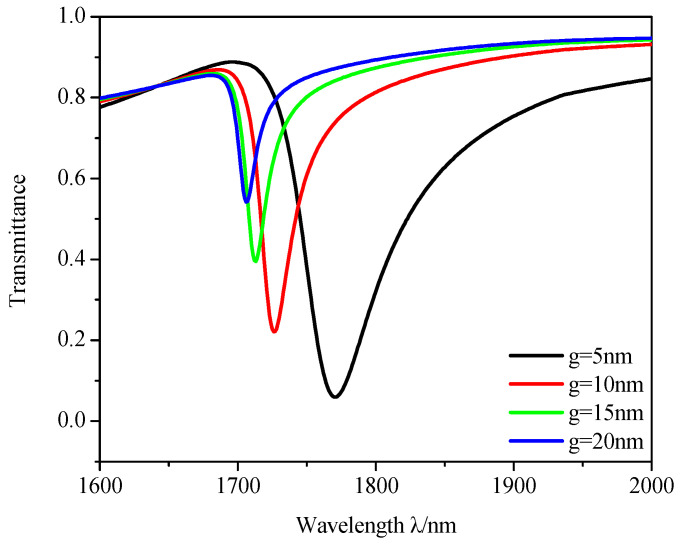
Transmission spectra of different coupling distances g.

Finally, we analyzed the effects of different coupling distances on sensing properties. The *g* was set from 5 to 20 nm and the results are plotted in Figure 10. With an increase in *g*, there is an obvious blueshift and the transmittance increases swiftly and FWHM becomes narrower, which means the confinement of the cavity to energy becomes weaker. Of course, the attenuation distance is $\delta_{Air}$ = 34 nm longer than the coupling distance. Therefore, we can infer that SPPs from the stub can be coupled into the racetrack ring resonator theoretically.

## 4. Conclusions

In this article, we propose a plasmonic nanosensor based on a metal-insulator-metal bus waveguide with a stub coupling with a racetrack ring resonator. The EIT-like and Fano resonances are excited in this system. The transmission properties of the design are investigated on COMSOL 5.4 by using the 2D-FEM. The optimal geometrical parameters *L*, *R*, *r*, *h*, *x* and *g* are 200, 160, 110, 60, 100 and 10 nm, and their sensitivity and FOM are 1774 nm/RIU and 61 when the FWHM is 29, respectively. The normalized H_Z_ field is highly affected by the displacement (*x*) of the ring from the stub. The sensing performance can be controlled by adjusting the geometric parameters. *R* and *L* can adjust the sensitivity significantly. *H* is the key to controlling the line shape and the FOM. The simple sensing structure we have proposed offers a better choice for nanosensing.

## Figures and Tables

**Table 1:** Comparisons of results with previous research.

Reference	Sensitivity	Structure	Operating Wavelength Range
This paper	1774 nm/RIU	Racetrack ring resonator	1200–1800 nm
[20]	1180 nm/RIU	Circular split-ring resonator	500–1200 nm
[21]	1350 nm/RIU	Ring resonator	700–1400 nm
[22]	1075 nm/RIU	Cross-shaped cavity and baffle	800–1300 nm
[23]	1200 nm/RIU	Isosceles triangular cavity	1000–1400 nm
[24]	1262 nm/RIU	Key-shaped resonator	600–1000 nm
[25]	2464 nm/RIU	Square ring resonator with nano-slit	1200–1800 nm
[26]	2080 nm/RIU	Nanoring resonator	500–2600 nm

## Data Availability

The available data has been stated in the article.
